# Prostate Health Index Density Outperforms Prostate Health Index in Clinically Significant Prostate Cancer Detection

**DOI:** 10.3389/fonc.2021.772182

**Published:** 2021-11-19

**Authors:** Shih-Ting Chiu, Yung-Ting Cheng, Yeong-Shiau Pu, Yu-Chuan Lu, Jian-Hua Hong, Shiu-Dong Chung, Chih-Hung Chiang, Chao-Yuan Huang

**Affiliations:** ^1^ Department of Urology, National Taiwan University Hospital, Taipei, Taiwan; ^2^ Department of Urology, National Taiwan University Hospital Hsin-Chu Branch, Hsinchu, Taiwan; ^3^ Division of Urology, Department of Surgery, Far-Eastern Memorial Hospital, New Taipei City, Taiwan; ^4^ Graduate Program in Biomedical Informatics, College of Informatics, Yuan-Ze University, Chung-Li, Taiwan; ^5^ Department of Urology, Taipei Veterans General Hospital, Yuan-Shan/Su-Ao Branch, Yi-Lan, Taiwan; ^6^ Department of Medical Research and Education, Taipei Veterans General Hospital, Yuan-Shan/Su-Ao Branch, Yi-Lan, Taiwan

**Keywords:** prostate health index density, risk table, clinically significant prostate cancer, save unnecessary prostate biopsy, predict lethal disease

## Abstract

**Background:**

Prostate-specific antigen (PSA) is considered neither sensitive nor specific for prostate cancer (PCa). We aimed to compare total PSA (tPSA), percentage of free PSA (%fPSA), the PSA density (PSAD), Prostate Health Index (PHI), and the PHI density (PHID) to see which one could best predict clinically significant prostate cancer (csPCa): a potentially lethal disease.

**Methods:**

A total of 412 men with PSA of 2–20 ng/mL were prospectively included. Serum biomarkers for PCa was collected before transrectal ultrasound guided prostate biopsy. PHI was calculated by the formula: (p2PSA/fPSA) x √tPSA. PHID was calculated as PHI divided by prostate volume measured by transrectal ultrasound.

**Results:**

Of the 412 men, 134 (32.5%) and 94(22.8%) were diagnosed with PCa and csPCa, respectively. We used the area under the receiver operating characteristic curve (AUC) and decision curve analyses (DCA) to compare the performance of PSA related parameters, PHI and PHID in diagnosing csPCa. AUC for tPSA, %fPSA, %p2PSA, PSAD, PHI and PHID were 0.56、0.63、0.76、0.74、0.77 and 0.82 respectively for csPCa detection. In the univariate analysis, the prostate volume, tPSA, %fPSA, %p2PSA, PHI, PSAD, and PHID were all significantly associated with csPCa, and PHID was the most important predictor (OR 1.41, 95% CI 1.15–1.72). Besides, The AUC of PHID was significantly larger than PHI in csPCa diagnosis (*p*=0.004). At 90% sensitivity, PHID had the highest specificity (54.1%) for csPCa and could reduce the most unnecessary biopsies (43.7%) and miss the fewest csPCa (8.5%) when PHID ≥ 0.67. In addition to AUC, DCA re-confirmed the clinical benefit of PHID over all PSA-related parameters and PHI in csPCa diagnosis. The PHID cut-off value was positively correlated with the csPCa ratio in the PHID risk table, which is useful for evaluating csPCa risk in a clinical setting.

**Conclusion:**

The PHID is an excellent predictor of csPCa. The PHID risk table may be used in standard clinical practice to pre-select men at the highest risk of harboring csPCa.

## Introduction

Prostate cancer (PCa) is one of the most common malignancies in both Western and Asian countries. The introduction of the prostate-specific antigen (PSA) test in 1987 is one of the reasons for the growing incidence of PCa. Produced by prostate epithelial cells, PSA is regarded as an organ-specific rather than a disease-specific marker. The correlation between PSA and benign prostate hyperplasia, prostate inflammation, and PCa makes it a marker with broad clinical utility; however, it is a complex tool in terms of confirming the cancer diagnosis, with a 60%–70% false positive rate (1–3).

About 2% of patients have post-biopsy complications, such as infection, bleeding, or voiding difficulty (3). Moreover, overdiagnosis of low-risk tumors possibly leads to overtreatment and the possibility of subsequent harm (4). Thus, when to perform a prostate biopsy should be individualized and well discussed.

Prostate Health Index (PHI), a novel PCa screening alternative, is calculated with total PSA (tPSA), free PSA (fPSA), and [-2]pro-PSA (p2PSA) using the following formula: (p2PSA/fPSA) x √tPSA. PHI is proved to be better at predicting the presence of PCa and its aggressiveness than tPSA, fPSA, and PSA density (PSAD) in multiple studies in both Western and Asian countries (5–9). Current guidelines suggest considering PHI testing before prostate biopsy to increase specificity and to avoid unnecessary biopsy (10).

In recent years, PHI density (PHID) has been a focus of research for its clinical utility. One prospective study of 118 men in Western society receiving prostate biopsy showed PHID is associated with clinically significant prostate cancer (csPCa) and outperformed PHI in the area under the receiver operating characteristic curve (AUC) analysis (11). PHID is found to predict cancer aggressiveness in post-radical prostatectomy pathologies, such as high-grade cancer or extracapsular prostatic invasion (12). CsPCa [defined as a Gleason score (GS) of 6 with ≥3 positive cores and/or a maximum core participation of ≥50%, or GS ≥7 as the Epstein criteria (13)] is a potentially lethal disease that requires early diagnosis and active treatment. However, very few studies discuss the role of PHID in detecting csPCa, or how many unnecessary prostate biopsies could be avoided with PHID. Thus, this study aims to evaluate the performance PHID in csPCa detection.

## Material and Methods

### Study Population

This single-center prospective study was conducted in line with National Taiwan University Hospital guidelines. The study was approved by the institutional review board at the National Taiwan University Hospital (approval code: 201612091RIPD), and informed consent was obtained from all individual participants included in the study. Initially, 542 consecutive men undergoing prostate biopsy for suspected PCa were enrolled in the study. Inclusion criteria were as follows, adult patients with a total PSA between 2 and 20 ng/ml or abnormal digital rectal examination (DRE), who received transrectal ultrasound guided prostate (TRUS-P) biopsy for at least systemic 12 cores at one single tertiary center between February 2017 and January 2020. Patients underwent TRUS-P biopsy with a standardized protocol for at least 12 biopsy cores (range: 12-22). Additional finger-guided biopsy was decided by the physicians if palpable prostate nodules.

Exclusion criteria were as follows: 1) patients with untreated urinary tract infection or bacterial acute prostatitis; 2) patients who had transurethral resection of the prostate previously; 3) patients with prior history of prostate cancer; 4) patients who were treated with 5-alpha reductase inhibitors, such as finasteride or dutasteride. A total number of 412 patients with written informed consent were included in the final analysis.

### Laboratory Analysis

After obtaining informed consent, blood samples were collected in ethylenediaminetetraacetic acid tubes before prostate biopsy and stored at -80°C after centrifugation. Serum samples were centrifuged at 1500 g for 15 min within 3 h of blood collection and stored at -20°C until analysis. The tPSA, fPSA, and p2PSA levels were analyzed with a Beckman Coulter Access 2 immunoassay analyzer (Beckman Coulter, Taiwan Inc.) with Beckman Coulter Access Hybritech reagent. The technology of chemiluminescent immunoenzymatic with Hybrithech PSA standardization was used. PHI was calculated according to the formula: PHI = (p2PSA/fPSA) x √tPSA. %fPSA was defined as (fPSA/tPSA) x 100; and %p2PSA was defined as [(p2PSA pg/mL)/(fPSA ng/mL x 1000)] x 100. Prostate volume was estimated with transrectal ultrasound with the standard ellipsoid formula: width x height x length x 0.52. PSA density (PSAD) was calculated with (tPSA/prostate volume), and PHI density (PHID) was calculated with (PHI/prostate volume).

Biopsy specimens were graded according to the updated Gleason grading system of the International Society of Urological Pathology (14). The specimens were examined by experienced genitourinary pathologists, who were blinded to the serum test results. csPCa Epstein criteria was defined as a Gleason score ≥7, or a Gleason score of 6 but with ≥3 positive cores and/or a maximum core involvement of ≥50% (13).

### Study End Points

The primary end point was to evaluate the sensitivity, specificity, diagnostic accuracy, and clinical benefit of %fPSA, PSAD, %p2PSA, PHI and PHID (index tests) in determining the presence of PCa and csPCa at prostate biopsy in comparison to tPSA (standard tests).

### Statistical Analysis

The primary outcome was csPCa found on biopsy. Continuous variables were reported as median and interquartile range (IQR). Statistical differences were assessed with Mann–Whitney U test for continuous variables and Chi-square test for categorical variables. Univariable logistic regression was used to determine the association between measured covariates and prostate cancer and clinically significant prostate cancer. The area under the receiver operating characteristic curve (AUC) was used to examine the diagnostic ability of each PSA derivative. Difference between AUCs were evaluated with DeLong test. Decision curve analysis (DCA) was applied to compare different diagnostic strategies with regards to maximizing clinical net benefit at different threshold probability (15). Statistical analyses were performed with SPSS version 22.0 (IBM Corp, Inc) and R software. A two-sided p value of <0.05 was considered significant.

## Results

Of the 412 men included, 134 (32.5%) were diagnosed with PCa and 57 (42.5%) had a GS of 6 PCa. 94 of 412 men (22.8%) were diagnosed with csPCa, of which 77 (81.9%) were GS ≥7 and the rest 17 men (18.1%) had a GS of 6 fulfilling the Epstein criteria. (**Table 1**). In the baseline characteristics, men with csPCa were significantly older, had a higher proportion of abnormal DRE, and a smaller prostate volume than the non-csPCa group. As regards biomarkers, the tPSA level was similar between the two groups, while men with csPCa had a significantly lower %fPSA, and higher %p2PSA, PHI, PSAD, and PHID.

**Table 1:** Characteristics of the study cohort.

	Overall (N = 412) (100%)		Benign (N = 278) (67.5%)		PCa (N = 134) (32.5%)		P value (*vs* benign)	csPCa (N = 94) (22.8%)		P value (*vs* non-csPCa)
Age, years	66	(60,71)	64	(58,69)	68	(63,74)	<0.001	68	(63,74)	<0.001
Abnormal DRE, n (%)	75	(18.2%)	33	(11.97%)	42	(31.34%)	<0.001	31	(32.9%)	<0.001
Prostate volume, ml	45	(34,61)	49	(38,67)	36	(27,47)	0.406	33	(25,43)	<0.001
Total PSA, ng/mL	7.2	(5.2,9.7)	7.2	(5.2,9.4)	7.4	(5.1,10)	0.002	7.7	(5.5,11.4)	0.064
%fPSA	16.6	(11.7,22.4)	17.6	(12.8,23.2)	14.3	(10.7,20.4)	<0.001	13.9	(10.1,19.1)	<0.001
%p2PSA	1.36	(1.01,1.81)	1.18	(0.94,1.57)	1.64	(1.36,2.16)	<0.001	1.73	(1.41,2.29)	<0.001
PHI	35.9	(25.8,47.9)	31.4	(24.6,42.2)	44.7	(34.5,58.6)	<0.001	47.8	(38.3,65.5)	<0.001
PSA density	0.15	(0.11,0.22)	0.14	(0.1,0.19)	0.20	(0.13,0.34)	<0.001	0.22	(0.15,0.39)	<0.001
PHI density	0.74	(0.48,1.32)	0.62	(0.41,0.95)	1.31	(0.78,2.01)	<0.001	1.49	(1.0,2.21)	<0.001
Gleason score, n(%) 3+3					57*	(42.5)		17	(18.1)	
3+4					39	(29.1)		39	(41.5)	
4+3					25	(18.7)		25	(26.6)	
8					6	(4.5)		6	(6.4)	
9					7	(5.2)		7	(7.4)	

PCa, prostate cancer; cs, clinically significant; DRE, digital rectal examination; PSA, prostate-specific antigen; %fPSA, percentage of free to total PSA; %p2PSA, percentage of p2PSA to free PSA ratio; PHI, Prostate Health Index; Data are median [interquartile range (IQR)] unless otherwise indicated.

*40 patients demonstrated insignificant PCa, and 17 patients demonstrated csPCa, according to the Epstein criteria.

The univariable logistic regression (**Table 2**) showed that age, abnormal DRE, and prostate volume were significant predictors for both PCa and csPCa. However, tPSA failed to demonstrate significance in predicting PCa (OR 1.04, P=0.129) but was a predictor for csPCa (OR 1.09, P=0.005). Contrarily, biomarkers such as %fPSA, %p2PSA, PHI, PSAD, and PHID were all significantly associated with both PCa and csPCa. The prostate volume factor plus PSA-related serum markers demonstrated that PSAD and PHID were the most important predictors of PCa (OR 1.42, 95% CI 1.21–1.67, and OR 2.27, 95% CI 1.73–2.97, respectively) and csPCa (OR 1.24, 95% CI 1.07–1.44, and OR 1.41, 95% CI 1.15–1.72, respectively).

**Table 2:** Univariable logistic regression models for the prediction of PCa and csPCa.

Variable	PCa		csPCa	
	Odds ratio (95%CI)	*P* value	Odds ratio (95%CI)	*P* value
Age	1.07 (1.04, 1.1)	<0.001	1.07 (1.04, 1.1)	<0.001
Abnormal DRE	3.39 (2.02, 5.67)	<0.001	3.06 (1.8,5.23)	<0.001
Prostate volume	0.96 (0.95, 0.97)	<0.001	0.94 (0.93,0.96)	<0.001
Total PSA	1.04 (0.99, 1.1)	0.129	1.09 (1.03-1.16)	0.005
%fPSA^*^	0.96 (0.93, 0.99)	0.004	0.94 (0.91,0.97)	<0.001
%p2PSA^*^	1.01 (1.01, 1.02)	<0.001	1.01 (1.01, 1.02)	<0.001
PHI	1.04 (1.03, 1.06)	<0.001	1.05 (1.03-1.06)	<0.001
PSA density^†^	1.42 (1.21, 1.67)	<0.001	1.24 (1.07,1.44)	0.005
PHI density	2.27 (1.73, 2.97)	<0.001	1.41 (1.15,1.72)	0.001

PCa, prostate cancer; cs, clinically significant; DRE, digital rectal examination; PSA, prostate specific antigen; %fPSA, percentage of free to total PSA; %p2PSA, percentage of p2PSA to free PSA ratio; PHI, Prostate Health Index; CI, confidence interval; *per unit change of 1 percent; ^†^per unit change of 0.1.

**Figure 1:**
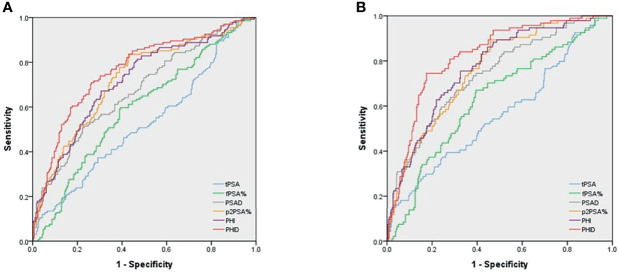
Area under the receiver operating characteristic (AUC) curves for predicting **(A)** PCa and **(B)** csPCa. **(A)** AUC for PCa detection were as follows: PSA 0.53, %fPSA 0.59, PSAD0.68, %p2PSA 0.72, PHI 0.72 and PHID 0.77, respectively. **(B)** AUC for csPCa detection were as follows: PSA 0.56, %fPSA 0.63, PSAD 0.74, %p2PSA 0.76, PHI 0.77 and PHID 0.82, respectively. The AUC diagnostic effect of PHID is still significantly better than PHI in PCa or csPCa (*p* = 0.007 and 0.004, respectively).

AUC was used to examine the ability of each diagnostic marker to indicate PCa (**Figure 1A**) and csPCa (**Figure 1B**). The predictors of PCa and csPCa in order from the worst to the best are as follows: tPSA (AUC= 0.53 and 0.56), %fPSA (AUC= 0.59 and 0.63), PSAD (AUC= 0.68 and 0.74), %p2PSA (AUC= 0.72 and 0.76), PHI (AUC= 0.72 and 0.77), and PHID (AUC= 0.77 and 0.82). The AUC of PHID was still significantly better than PHI in PCa or csPCa diagnosis (*p*=0.007 and 0.004, respectively). Among the tested biomarkers, PHID showed the highest discriminative ability for PCa and csPCa.

With a 90% sensitivity for detecting csPCa, PHID had the highest specificity at 54.1%, while tPSA only demonstrated a specificity of 17.9% (**Table 3**). At the cut-off value of ≥0.67 for PHID, it could reduce the most unnecessary biopsies (43.7%) and missed the least cases of csPCa (8.5%). On the other hand, at the given cut-off values with tPSA of ≥4.43 ng/mL, %fPSA ≤0.26, %p2PSA ≥1.12, PHI ≥31.0, and PSAD ≥0.11 ng/mL/cc, the avoidable biopsy percentages were 15.5%, 14.8%, 32.8%, 37.4% and 26.9% respectively. In summary, PHID is the best marker for csPCa in all PSA-related parameters.

**Table 3:** Specificity, reduction of unnecessary biopsy, and missing positive cases at 90% sensitivity at predicting csPCa.

Biomarkers	Cut-off value	Specificity (%)	Avoidable biopsies (% of all biopsies, N = 412)		Missed biopsies (% of csPCa, N = 94)	
Total PSA	≥4.43	17.9%	64	(15.5%)	9	(9.6%)
%fPSA	≤0.26	16.7%	61	(14.8%)	10	(10.6%)
%p2PSA	≥1.12	39.9%	135	(32.8%)	9	(9.6%)
PHI	≥31.0	45.3%	154	(37.4%)	10	(10.6%)
PSA density	≥0.11	31.8%	111	(26.9%)	10	(10.6%)
PHI density	≥0.67	54.1%	180	(43.7%)	8	(8.5%)

csPCa, clinically significant prostate cancer; PSA, prostate specific antigen; %fPSA, percentage of free to total PSA; %p2PSA, percentage of p2PSA to free PSA ratio; PHI, Prostate Health Index.

**Figure 2:**
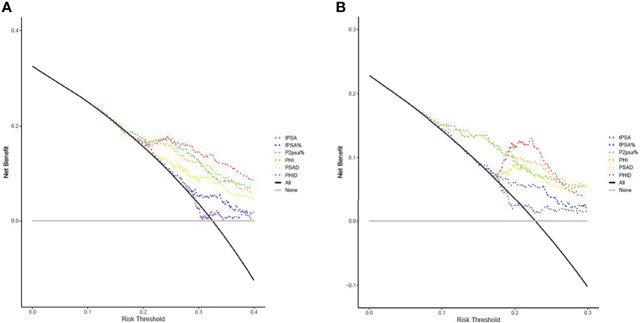
Decision curve analysis (DCA) of various models on **(A)** PCa detection and **(B)** csPCa detection in comparison to biopsy-all (black curve line) and biopsy-none strategies (grey horizontal line). The markers of the best clinical benefit in the diagnosis of PCa and csPCa are list in order as follows: PHID, PHI, %p2PSA, PSAD, %fPSA and tPSA. Model of PHID (red dotted line) resulted in greater net benefit in overall PCa and csPCa detection at probability threshold range 20% to 30%.

DCA is an analytic method for comparing different diagnostic strategies with regards to maximizing clinical net benefit against different given threshold probability. DCA curves for different biopsy scenarios indicated by various PSA-related parameters, PHI and PHID were plotted in **Figure 2**. The models of each biomarker for csPCa diagnosis were listed in the order from the most net benefit to the least as follows: PHID, PHI, %p2PSA, PSAD, %fPSA and tPSA at probability threshold range 20% to 30% (**Figure 2B**). As for the diagnosis of PCa (**Figure 2A**), there was an order almost similar to that of csPCa. Consistently with the AUC results, PHID had the most improvement in clinical net benefit at initiating biopsy for both PCa and csPCa.

In addition, a risk table was made to evaluate the positive chances of PCa, csPCa and high-grade PCa (GS ≥7) under different PHID values (**Table 4**). We found the PHID cut-off value was positively correlated with the PCa, csPCa, and HGPCa ratio. For the PHID cut-off value of 0.5–0.75, the risks of PCa, csPCa, and HGPCa were 17.2%, 10.1%, and 7.1%, respectively. If an individual had a PHID value of 1–1.5, it was assumed that they had about a one-third chance of having csPCa; if the PHID value was over 1.5, they had a nearly 50% chance of having csPCa. In summary, the PHID risk table may be used in standard clinical practice to pre-select men at the highest risk of harboring csPCa.

**Table 4:** Percentage of PCa, csPCa, and high-grade PCa (HGPCa) diagnosed at different PHI density values.

	PHI density cut-off value					Total
	<0.5	0.5-0.75	0.75-1	1-1.5	>1.5	
PCa	12.8% (14/109)	17.2% (17/99)	30.9% (17/55)	48.5% (32/66)	65.1% (54/83)	32.5% (134/412)
csPCa	3.7% (4/109)	10.1% (10/99)	16.4% (9/55)	37.9% (25/66)	55.4% (46/83)	22.8% (94/412)
HGPCa (GS≥7)	2.8% (3/109)	7.1% (7/99)	12.7% (7/55)	28.8% (19/66)	49.4% (41/83)	18.7% (77/412)

PHI, Prostate Health Index; PCa, prostate cancer; cs, clinically significant; HG, high-grade; GS, Gleason score.

## Discussion

In our prospective cohort, we compared the performance of tPSA, %fPSA, %p2PSA, PSAD, PHI, and PHID in terms of predicting csPCa without missing the diagnosis. We found PHID was the best predictor of csPCa and could greatly reduce the number of unnecessary biopsies. We also found that the PHID cut-off value was positively correlated with the ratio of csPCa. We could further evaluate the patient’s csPCa risks based on this PHID risk table to decide whether to arrange a prostate biopsy. In our understanding, this is the first time a PHID risk table to evaluate the csPCa risk has been established.

PSA is neither sensitive nor specific in csPCa prediction, leading to many unnecessary biopsies and indolent cancer detected (1–3). There are several proteomic and genomic tools being studied to better diagnose csPCa, including PHI, 4K score and Stockholm3 as blood tests, and Mi-prostate score, Exo DX Prostate, and Select MD-X as urinary biomarker-based tests (16). Besides, liquid biopsy using circulating tumor cells (CTC) play an emerging and promising role in genitourinary oncology (17, 18). CTC may act as tools for pre-diagnosis screening, post-diagnosis risk stratification, and treatment response evaluation in PCa (19, 20).

There is no denying that multiparametric magnetic resonance imaging (mpMRI) is the best tool for predicting csPCa (21–23), but mpMRI may not be the case in terms of cost-effectiveness. We try to make a trade-off between diagnostic accuracy and cost-effectiveness among these tests and examinations. In previous study, mpMRI could indeed provide higher diagnostic accuracy in identifying csPCa than PHI (23). However, there are high rates of interobserver disagreements in reading prostate MRIs between different radiologists (24). mpMRI is also a resource-intensive and time-consuming examination. The costs of a prostate MRI are estimated to be €300–€500 in Europe and $700–$3000 in regions outside of Europe (25). In terms of cost-effectiveness, Kim et al. suggest that PHI as a triaging test may be an effective way to reduce mpMRI and biopsies without compromising the detection of csPCa (26). The cost-effectiveness of PHI testing is explored in both Western and Eastern world. The PHI-based strategy is more cost-effective than the PSA-based strategy for PCa regardless of what willingness-to-pay threshold by reducing biopsy costs and biopsy-related adverse events (27–30). The results may be applied not only in developed regions but also in developing countries (31).

To make up for the shortcomings of the low specificity and low sensitivity of PSA, PHI was developed. The first prospective PCa screening study in 2010 found PHI and %p2PSA (AUC= 0.77 and 0.76) could distinguish PCa from benign diseases more accurately than tPSA (AUC= 0.50) (32). Afterwards, many studies (5–8, 33–36) and our previous study (9) found that using PHI would detect PCa more accurately than tPSA, avoiding a considerable degree of unnecessary prostate biopsies. More importantly, PHI has shown promise in being able to differentiate csPCa more accurately from clinically insignificant PCa than tPSA, improving PCa cancer death rates and reducing unnecessary overdiagnoses and overtreatment of insignificant PCa (37). Furthermore, Fossati et al. (38) and our previous study (39) found that PHI can significantly improve the prediction of unfavorable PCa characteristics, larger tumor volume, and csPCa at final radical prostatectomy pathology (40).

PHI shows an excellent ability to accurately diagnose csPCa in different races. Our previous study (7) shows that for PSA 2–10 ng/ml, when we set the PHI threshold to 35, the PCa positive rate of Europeans and Asians can be increased from 52.1% and 13.1% to 66.6% and 29.4%, respectively. More importantly, in both Europeans and Asians, we can diagnose GS≥7 PCa more accurately, which increased from 28.8% and 8.1% to 40.2% and 21.5%, respectively. PHI (cut-off 35) can help avoid 32.6% and 71.1% of unnecessary biopsies in Europeans and Asians. In summary, although the PHI threshold of different races should be adjusted, the excellent diagnostic ability of PHI is the same.

Larger prostate volume is associated with increased PSA levels (41). Benson et al. first demonstrated that PSAD helped differentiate between benign prostate hypertrophy and PCa in PSA levels 4–20 ng/mL (42). Numerous following studies had similar results of the PSAD superiority over PSA in detecting PCa and adverse pathology (43–46). Similar to the conclusions of other articles, we found that PSAD was one of the top predictors of csPCa. PSAD also improved the diagnostic accuracy in patients with Prostate Imaging Reporting & Data System (PI-RADS) score ≤3 lesions in MRI, and the combination of PSAD and MRI was advocated to individualize prostate biopsy strategy (47–49).

Prostate volume is an important factor for csPCa and should be added to PSA-related factors to improve PCa detection. Filella et al. found PHI to be associated with prostate volume. The AUCs of PHI in patients with small, medium, and large prostate volumes were 0.818, 0.716, and 0.654, respectively, suggesting that a larger prostate size would decrease PHI diagnostic ability (50). Recently, studies have found that PHID is more significantly related to csPCa than other PSA-related parameters. In a prospective study by Tosoian et al., which consisted of 118 men with PSA>2 ng/mL and negative DRE, the median PHID value was 0.70 in the negative biopsy group, 0.53 in the clinically insignificant PCa group, and 1.21 in the csPCa group (p< 0.001). A higher PHID value is also significantly associated with more csPCa (3.6%, 36.7%, and 80.0% csPCa in PHID <0.43, 0.43–1.21, and >1.21, respectively, p<0.001). PHID was found to have the highest discriminative ability to detect csPCa (AUC 0.84) as compared to PSA, PSAD, %fPSA, and PHI. Moreover, PHID could be used to avoid 38% of unnecessary biopsies, while failing to detect only 2% of csPCa cases (11). Likewise, Barisiene et al. demonstrated that PHID best detected csPCa (AUC 0.80) and could help avoid 30% of prostate biopsies (51). Schulze et al. showed that PHID had a better performance in predicting PCa than PHI, PSAD, %fPSA, and tPSA. Only one csPCa case would have been missed in 50 csPCa cases (sensitivity 98%), and 20% of prostate biopsies could have been avoided with a combined use of PHID >0.9 and PHI >40 (52). Garrido et al. found PHID had the highest AUC in predicting overall PCa and csPCa (AUC 0.82 and 0.85, respectively) but there were no significant differences between the AUCs of PHID and PHI or between PHID and PSAD (53). A large retrospective cohort study demonstrated that PHID had similar AUC as PHI and had a small advantage on decision curve analysis than PHI alone in predicting overall PCa (54). Stephan et al. found PHID had significantly larger AUC than PHI in predicting overall PCa but no significant difference from PHI if aiming for csPCa (55). In our study, the AUC of PHID is significantly better than PHI in predicting csPCa (*p*=0.004, **Figure 1B**). Our study concluded that PHID is the best predictor of PCa and csPCa among various PSA-related biomarkers consistently both with AUC analysis and DCA.

The optimal PHID cut-off value was still not determined in as a result of the scarcity of the related studies. Tosoian et al. proposed a cut-off value 0.43 to detect Epstein significant disease with a 97.9% sensitivity and a 38.0% specificity (11). Barisiene et al. suggested a cut-off value of 0.61 to detect Epstein significant PCa at a 90% sensitivity, which had a resemblance to our PHID cut-off of 0.67 (51). Garrido et al. proposed PHID ≥ 0.49 as cut-off for csPCa, sparing 26.3% biopsies at 90% sensitivity (53). Besides, in men with initial negative prostate biopsy, those with initial PHID≥ 1.2 may have 21% risk developing csPCa at 6-year follow up, while those with PHID <0.4 had lowest risk and may not need intensive follow-up, depicted in a recent study by Liu et al. (56). In our study, at 90% sensitivity, PHID had the highest specificity (54.1%) for csPCa and could reduce the most unnecessary biopsies (43.7%) and miss the least csPCa (8.5%) when PHID > 0.67 (**Table 3**). We constructed a comprehensive table consisting of different PHID ranges and the corresponding risk for both PCa and csPCa (**Table 4**); for instance, the median PHID was 0.62 (0.41–0.95), 1.31 (0.78–2.01), and 1.49(1.0–2.21) in men with negative biopsy, PCa, and csPCa (p<0.001). The risk values for csPCa were 3.7%, 20.0%, and 55.4% for PHID <0.50, 0.5–1.5, and >1.50, respectively. We can avoid 43.7% of unnecessary biopsies and only miss 8.5% of csPCa cases for PHID > 0.67. The PHID and PHID risk tables may be used in standard clinical practice to pre-select men at a higher risk of harboring csPCa.

Association of different PCa biomarkers with mpMRI findings is another interesting topic worth researching. The combination of biomarkers and mpMRI may result in more clinical benefit than PSA plus mpMRI, especially in those who had equivocal PI-RADS scores (49, 57, 58). Druskin et al. recommended that prostate biopsy be performed in patients with PI-RADS ≥3 lesions in MRI or PHID ≥ 0.44 if PI-RADS score ≤2; this was 100% sensitive for csPCa detection (59). Similarly, in patients with at least one PI-RADS≥ 3 lesion in MRI, PHID added the greatest diagnostic value when fusion targeted biopsy methods were performed (60). We believe that the incorporation of PHID and MRI findings is a promising avenue and warrants further larger scale studies.

Our study had several limitations. First, the study cohort was heterogenous with biopsy-naïve subjects and those with biopsy histories. This may have confounded the results, as less PCa was detected in those who had prior biopsies. Secondly, the sample size of our study was relatively small. The PHID results and PHID risk table for csPCa need further external verification by other studies focused on different ethnicities. Third, mpMRI was not routinely performed in our entire cohort. We are incorporating PHI and mpMRI data from our cohort database. Further study results will be analyzed when more subjects are enrolled in the coming future.

## Conclusions

In our prospective cohort, we found that PHID had the best performance, could reduce the most unnecessary biopsies, and missed the fewest csPCa cases. The PHID cut-off value is positively correlated with the csPCa ratio in the PHID risk table. In conclusion, the PHID has excellent ability to predict csPCa before biopsy. The PHID risk table may be used in standard clinical practice to pre-select men at a higher risk of harboring csPCa.

## Data Availability Statement

The original contributions presented in the study are included in the article/supplementary material. Further inquiries can be directed to the corresponding authors.

## Ethics Statement 

The studies involving human participants were reviewed and approved by National Taiwan University Hospital (approval code: 201612091RIPD). The patients/participants provided their written informed consent to participate in this study. Written informed consent was obtained from the individual(s) for the publication of any potentially identifiable images or data included in this article.

## Author Contributions

Study design and conceptualization: C-YH, C-HC, Y-TC. Data collection: Y-TC and S-TC. Formal analysis: Y-CL, J-HH, and S-DC. Writing—original draft preparation: Y-TC and S-TC. Writing—review and editing: Y-SP, C-YH, and C-HC. All authors contributed to the article and approved the submitted version.

## Conflict of Interest

The authors declare that the research was conducted in the absence of any commercial or financial relationships that could be construed as a potential conflict of interest.

## Publisher’s Note

All claims expressed in this article are solely those of the authors and do not necessarily represent those of their affiliated organizations, or those of the publisher, the editors and the reviewers. Any product that may be evaluated in this article, or claim that may be made by its manufacturer, is not guaranteed or endorsed by the publisher.
